# Association between left ventricular systolic function parameters and myocardial injury, organ failure and mortality in patients with septic shock

**DOI:** 10.1186/s13613-023-01235-5

**Published:** 2024-01-18

**Authors:** Patrik Johansson Blixt, Maxime Nguyen, Bernard Cholley, Fredrik Hammarskjöld, Alois Toiron, Belaid Bouhemad, Shaun Lee, Lina De Geer, Henrik Andersson, Meriam Åström Aneq, Jan Engvall, Michelle S. Chew

**Affiliations:** 1 Department of Anaesthesiology and Intensive Care, Biomedical and Clinical Sciences, Linköping University, S-58185 Linköping, Sweden; 2 Department of Anaesthesiology and Intensive Care, Dijon University Hospital, Dijon, France; 3 Department of Anaesthesiology and Intensive Care Medicine, Hôpital Européen Georges Pompidou, Paris, France; 4 UMR-S1140 “Innovations Thérapeutiques en Hémostase”, Université Paris Cité, INSERM, Paris, France; 5 Department of Anaesthesia and Intensive Care, Ryhov County Hospital, Jönköping, Sweden; 6 Intensive Care Unit, St Georges Hospital, London, UK; 7 Department of Clinical Physiology, Biomedical and Clinical Sciences, Linköping University, Linköping, Sweden

**Keywords:** Septic shock, Left ventricle, Systolic function, Myocardial injury, Organ failure, Strain, MAPSE

## Abstract

**Background:**

Left ventricular ejection fraction (LVEF) is inconsistently associated with poor outcomes in patients with sepsis. Newer parameters such as LV longitudinal strain (LVLS), mitral annular plane systolic excursion (MAPSE) and LV longitudinal wall fractional shortening (LV-LWFS) may be more sensitive indicators of LV dysfunction, but are sparsely investigated. Our objective was to evaluate the association between five traditional and novel echocardiographic parameters of LV systolic function (LVEF, peak tissue Doppler velocity at the mitral valve (s´), LVLS, MAPSE and LV-LWFS) and outcomes in patients admitted to the Intensive Care Unit (ICU) with septic shock.

**Methods:**

A total of 152 patients admitted to the ICU with septic shock from two data repositories were included. Transthoracic echocardiograms were performed within 24 h of ICU admission. The primary outcome was myocardial injury, defined as high-sensitivity troponin T ≥ 45 ng/L on ICU admission. Secondary outcomes were organ support-free days (OSFD) and 30-day mortality. We also tested for the prognostic value of the systolic function parameters using multivariable analysis.

**Results:**

LVLS, MAPSE and LV-LWFS, but not LVEF and s´, differed between patients with and without myocardial injury. After adjustment for age, pre-existing cardiac disease, Simplified Acute Physiology (SAPS3) score, Sequential Organ Failure Assessment (SOFA) score, plasma creatinine and presence of right ventricular dysfunction, only MAPSE and LV-LWFS were independently associated with myocardial injury. None of the systolic function parameters were associated with OSFD or 30-day mortality.

**Conclusions:**

MAPSE and LV-LWFS are independently associated with myocardial injury and outperform LVEF, s´ and LVLS. Whether these parameters are associated with clinical outcomes such as the need for organ support and short-term mortality is still unclear.

*Trial registration* NCT01747187 and NCT04695119.

**Supplementary Information:**

The online version contains supplementary material available at 10.1186/s13613-023-01235-5.

## Background

Septic cardiomyopathy (SCM) occurs commonly among critically ill patients with sepsis, with prevalences ranging from 20 to 65% [1, 2]. Patients with SCM have a 2–3 times increased risk of mortality [1, 2]. Although no consensus exists for the definition of SCM, sepsis affects the systolic and diastolic functions of both ventricles [1, 2]. Investigations of left ventricular systolic function account for a majority of the reported literature. The most widely used echocardiographic definition for SCM is LVEF < 50%, however other left ventricular (LV) systolic function parameters have been proposed, such as peak systolic tissue Doppler velocity measured at the mitral annulus (s´), LV longitudinal strain (LVLS), mitral annular plane systolic excursion (MAPSE), and LV longitudinal wall fractional shortening (LV-LWFS). S´ does not appear to be related to mortality [3], although recent findings indicate that a more complex relationship may exist for LVEF and LVLS [4, 5]. The prognostic importance of systolic LV longitudinal function is increasingly recognised as an early indicator of adverse outcomes in patients with diverse heart diseases [6–8] with a majority of studies investigating LVLS. Available studies in patients with sepsis suggest that LVLS may be a more sensitive indicator of systolic dysfunction and an association with mortality was demonstrated in a recent meta-analysis [9] that was based on a limited number of heterogenous studies with several reporting contradictory results [10, 11]. In a recent study, LV-LWFS was proposed as a bedside surrogate measurement for LVLS in critically ill patients [12]. LV-LWFS is strongly correlated with LVLS in critically ill patients, a finding that was recently validated in patients with septic shock [13, 14]. Despite their simplicity and feasibility, LV-LWFS and its related parameter MAPSE, have been sparsely investigated in critically ill patients with few studies investigating their association with clinical outcome. Two studies in patients with sepsis have demonstrated the independent association between MAPSE and short-term mortality [15, 16]. However, these data were collected in a limited number of patients and only adjusted for a limited number of confounders.

Current definitions of SCM that are based on a reduced LVEF alone may be too simplistic and demonstrable myocardial injury seems to be a logical prerequisite to support the concept of a ‘myopathy’. While it may be intuitive that systolic dysfunction is associated with myocardial injury, defined as elevated high-sensitivity Troponin T (hsTnT) levels, it is poorly investigated among patients with septic shock. It is also unknown how specific echocardiographic markers of LV systolic function are related to the need for organ support and if they confer additional value when assessed in the presence of potential confounders.

Thus, the aim of this study is to evaluate the independent association between five echocardiographic measurements of LV systolic function; LVEF, s´, LVLS, MAPSE and LV-LWFS, with myocardial injury, organ support-free days (OSFD) and 30-day all-cause mortality.

## Methods

This study is a retrospective analysis of prospectively collected data for the explicit purpose of examining the relationship between echocardiographic parameters, biomarkers and outcomes. The dataset was drawn from the data repositories of two studies of patients admitted to intensive care units (ICU) for septic shock. All 177 patients from the original studies were assessed and 152 were included in the present study, 44 from the single-centre Septic Heart (SH) study (NCT01747187) and 108 from the multi-centre Sepsis in the ICU-2 (SICU-2) study (NCT04695119). Both studies were prospective, observational studies including patients with septic shock. Patients was included between 2012 and 2014 and 2019–2022 for the SH and the SICU-2 studies, respectively. Accordingly, SH included patients defined according to the Sepsis-II criteria, whilst SICU-2 recruits patients fulfilling the Sepsis-III criteria. The SICU-2 study is ongoing and currently active in four ICUs in Sweden and France. Written, informed consent was obtained from participants or their proxies. Exclusion criteria were < 18 years of age or lack of informed consent, and presence of acute coronary syndromes on admission (for SICU-2 only). Ethical approval was obtained for each study (Dnr. 2012/233-31, 2016/361-31 Linköping, Dossier 21.02984.000034—ID RCB: 2021-A02403-38 Dijon). A blinded observer not involved in patient care extracted data from the patient’s record using a predefined template. Baseline characteristics including comorbidities, pre-existing cardiac disease (defined as arrhythmia, heart failure or ischaemic heart disease), pre-existing medications, intensive care treatment, laboratory variables and outcomes were registered. Data were documented for the first seven days from ICU admission, or until discharge.

**Fig. 1:**
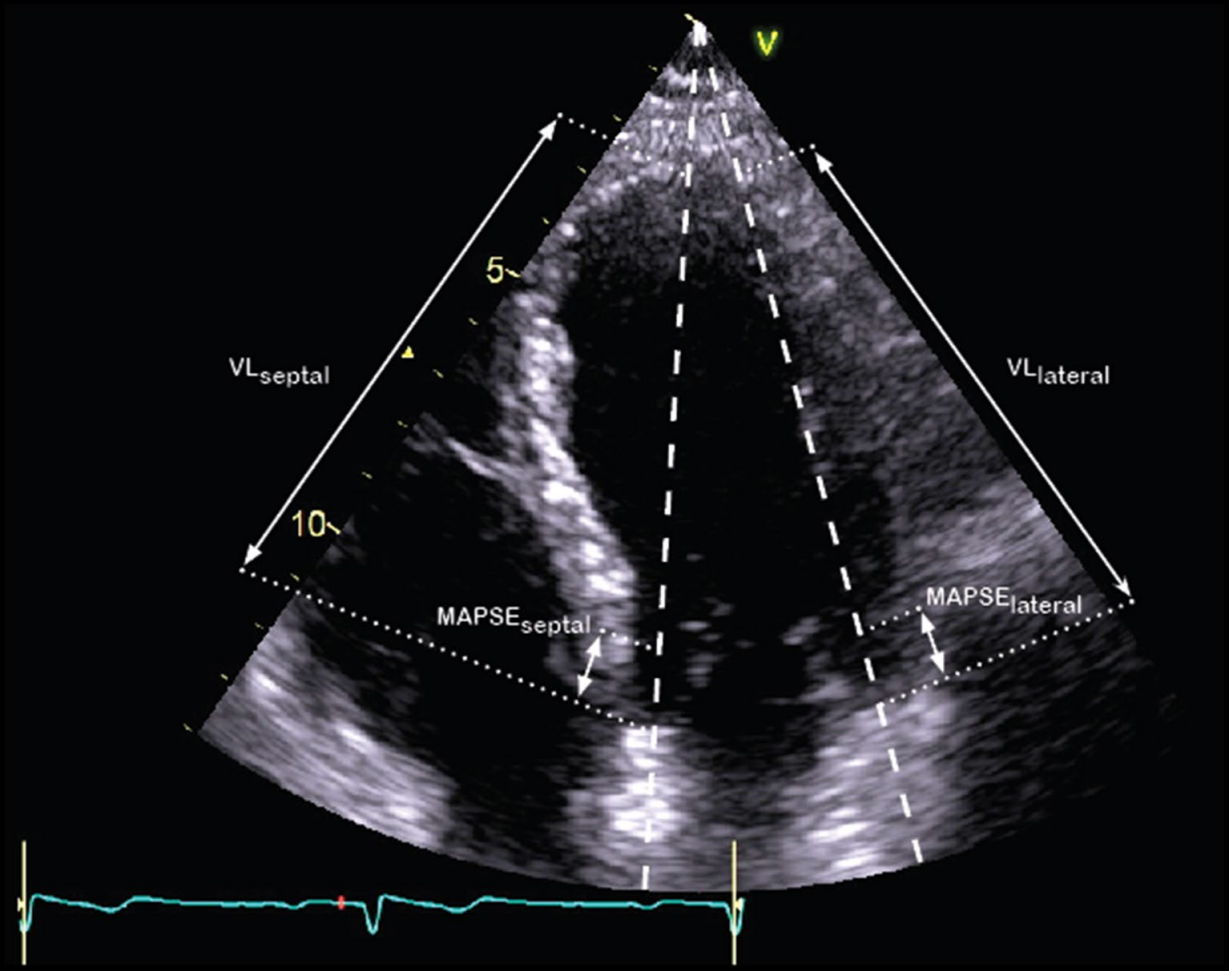
Measurement of MAPSE and VL

The TTE examinations were performed on a GE Vivid E9 or E95 scanner with a M5S-D transducer or a Philips Affiniti 70G with a S5-1 or X5-1 transducer, or a Philips CX-50 with a S5-1 transducer, or a Siemens SC2000 with a 4V1c transducer. All TTE examinations were conducted within 24 h of ICU admission by an experienced, certified physician, sonographer or a clinical physiologist. For patients on invasive mechanical ventilation, the tidal volume was set to 6–8 ml/kg of predicted body weight and echocardiographic recordings were made irrespective of the phase of respiration. We endeavoured to capture at least three and five heartbeats in patients with sinus rhythm and atrial fibrillation, respectively, for analysis. Recorded values for each variable is the average of these beats. RawDICOM and DICOM images (37 patients) from all sites were transferred to the central echocardiography laboratory at Linköping University Hospital for analysis. Images were imported and analysed in ViewPoint (GE Healthcare GmbH, Solingen, Germany) with EchoPAC Suite (GE EchoPAC plugin v. 202 and v. 203, GE Vingmed Ultrasound AS, Horten, Norway). Three experienced, certified assessors screened all images for suitability of analysis and performed measurements blinded to each other and to the clinical data. LVEF was measured using Simpson’s biplane method. Tissue Doppler s´ was measured and averaged from the septal and lateral basal walls in the A4C view. LVLS was measured using speckle tracking (Automated Function Imaging with individual optimisation) from the A4C view. Images had to be of sufficient quality for endocardial border tracing and have a frame rate-to-heart rate ratio of at least 0.5. This ratio was chosen by extrapolating recommendations of a minimum of 40 frames per second (FPS) and an estimated resting heart rate of 80 beats/min in a normal population [17]. Peak myocardial strain for each of the three lateral as well as the three medial segments was averaged. The average of septal and lateral MAPSE was calculated and measured in the A4C view. Post-systolic shortening was avoided by gating the measurements to the electrocardiogram. Ventricle length (VL) was measured in A4C, length calibration was performed for each image (Fig. 1). VL was calculated as the average of septal and lateral lengths. This method is easy to learn and requires little prior experience to obtain robust measurements as long as a proper A4C view is obtained. LV-LWFS was calculated according to the method by Huang et al. [12] and is given by Eq. 1.

LV-LWFS calculation.

1 $$LV-LWFS= \frac{MAPSE}{VL}\times 100.$$

RV systolic dysfunction was defined as free wall longitudinal strain (FWS) > -20%, tricuspid annular plane systolic excursion (TAPSE) < 17 mm, fractional area change (FAC) < 35%, peak systolic tissue or colour Doppler velocity measured at the tricuspid annulus (s´) < 9.5 cm/s or < 6.0 cm/s [18], respectively, or right ventricle to left ventricle (RV/LV) area ratio > 0.66 with concurrent paradoxical septal motion.

Although the majority of TTE examinations predated the PRICES statement, we endeavoured to report our findings accordingly [19].

For measurement of myocardial injury, we used an hsTnT assay. EDTA-plasma was collected from patients within six hours of ICU admission. Measurements were made in batch by technicians blinded to the results of the echocardiography findings and vice versa using automated immunoassays (Cobas e411 or e610, Roche Diagnostics GmBH, Mannheim, Germany) by a central chemistry lab accredited according to ISO-IEC 17025. The measuring range was 3–10000 ng/L and the 99th percentile (upper reference limit) is 14 ng/L in healthy individuals, with a total coefficient of variation of approximately 3.5%.

The primary outcome was myocardial injury, defined as an increased hsTnT ≥ 45 ng/L on ICU admission, with or without ischaemic symptoms. This definition was based on estimations from previous findings in ICU patients [15, 16] and corresponds to approximately three times the upper reference limit in a normal population. Secondary outcomes were OSFD and 30-day all-cause mortality. OSFD was defined as the number of days alive and without vasopressors/inotropes, invasive mechanical ventilation or renal replacement therapy within 30 days of study enrolment. Only days free from all organ-support therapies were counted as OSFD. OSFD was assigned a value of ‘0’ for patients dying within the observation period [20]. The vasoactive-inotropic score (VIS) was used to assess cardiovascular dysfunction [21]. The score is the mean VIS during admission day and calculated according to Eq. 2.

Vasoactive-inotropic score.

2 $$Dopamine\, dose \,(\mu g/kg/min)\,+\,dobutamine\, dose \,(\mu g/kg/min)\,+\,100\, x\, epinephrine \,dose \,(\mu g/kg/min),+10 \,x\, milrinone\, dose\, (\mu g/kg/min)\,+\,10 000\, x \,vasopressin \,dose\, (U/kg/min)\,+\,100\, x \,norepinephrine \left(base\right)\, dose\, (\mu g/kg/min).$$

## Statistics

Data are presented as median and interquartile range (IQR) for continuous variables and frequencies and percentages (%) for categorical variables. For comparisons, Mann–Whitney U-test was used for continuous data and Chi^2^-test for categorical data. Missing data were deleted listwise. Each echocardiographic variable was modelled in separate logistic or linear regressions to avoid possible collinearity. The multivariable models were adjusted for age, SAPS III score, pre-existing cardiac disease, and admission values of SOFA score and plasma creatinine concentrations. These covariates were chosen a priori based on previous findings and clinical plausibility. We reasoned that a robust multivariable analysis would require adjustment for 5–10 key independent variables. Using a rule of thumb of 10 outcomes per independent variable, we calculated that 70 outcomes were required to correct for 7 independent variables. Our sample size was based on an assumed frequency of myocardial injury of at least 50% [15, 16]. Given this assumption, a sample size of at least 140 patients was required.

A sensitivity analyses was conducted including patients without atrial fibrillation at the time of echocardiography. Interobserver variability was measured with the intraclass correlation coefficient.

All analyses were made in IBM SPSS Statistics (version 28.0, IBM Corp., Armonk, NY).

## Results

**Fig. 2:**
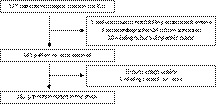
Inclusion and exclusion flowchart

A flowchart of inclusion and exclusion is shown in Fig. 2.

A total of 152 patients were finally included in the study. 76 patients (50%) were identified as having myocardial injury on ICU admission.

A large proportion (39%) of patients had pre-existing cardiac disease or any combination of these. Patients with myocardial injury were generally older with pre-existing cardiac disease and had a higher plasma creatinine and VIS on admission, compared to patients without myocardial injury (Table 1). Compared to the SH cohort, patients in the SICU-2 cohort had higher BMI, lower SOFA score, lower admission Hb, higher admission creatinine and a higher frequency of inotropes, but were otherwise similar (Additional file 1: Table S1).

**Table 1:** Baseline characteristics and biomarker findings on admission for all patients and stratified by myocardial injury

	All *n* = 152	No myocardial injury *n* = 76	Myocardial injury *n* = 76
Age years (IQR)	70 (58–76)	63 (54–73)	72 (62–78)
Sex, male *n* (%)	84 (55)	43 (61)	38 (50)
Body mass index kg/m^2^ (IQR), *n* = 133	27.7 (23.7–32.2)	27.3 (23.7–30.9)	28.9 (23.9–33.5)
Pre-existing cardiac disease† *n* (%)	59 (39)	22 (31)	36 (47)
SOFA score (IQR)	9 (7–12)	9 (7–12)	10 (8–13)
SAPS 3 core (IQR)	67 (57–76)	65 (55–76)	71 (62–77)
Clinical Frailty Scale score (IQR) *n* = 108	3 (2–4)	3 (2–4)	3 (3–4)
Haemoglobin g/L (IQR)	100 (84–112)	102 (85–113)	97 (82–109)
Creatinine μmol/L (IQR)	148 (98–225)	128 (82–184)	179 (120–234)
Lactate mmol/L (IQR)	3.2 (1.9–4.8)	3.3 (1.8–4.9)	3.2 (2.1–4.7)
hsTnT ng/L (IQR), *n* = 142	49 (23–98)	23 (15–37)	95 (62–233)
VIS score (IQR), *n* = 105	9.9 (4.2–15.8)	7.0 (3.5–12.6)	11.2 (4.6–19.0)
Inotropes‡ *n* (%)	64 (42)	27 (38)	35 (46)
Non-surgical admission cause n (%), *n* = 108	60 (56)	26 (55)	30 (54)
Surgical admission cause n (%), *n* = 108	48 (44)	21 (45)	26 (46)
Acute surgery *n* (%)	42 (87)	19 (91)	22 (85)
Elective surgery *n* (%)	6 (13)	2 (9)	4 (15)
Sepsis source, *n* = 108			
Abdominal *n* (%)	32 (30)	15 (32)	16 (29)
Urological/kidneys *n* (%)	27 (25)	10 (21)	15 (27)
Lungs/airways *n* (%)	21 (19)	10 (21)	9 (16)
Muscle/fascia *n* (%)	10 (9)	7 (15)	3 (5)
Hepatobiliary/pancreas *n* (%)	6 (6)	1 (2)	5 (9)
Catheter related *n* (%)	6 (6)	1 (2)	3 (5)
Skin *n* (%)	3 (3)	1 (2)	2 (4)
Other *n* (%)	2 (2)	1 (2)	1 (2)
Unknown *n* (%)	1 (1)	1 (2)	0 (0)

Myocardial injury defined as high-sensitivity troponin T ≥ 45 ng/L on ICU admission

*SOFA* Sequential Organ Failure Assessment. *SAPS 3* Simplified Acute Physiology Score 3. HsTnT: high-sensitive Troponin T. *VIS* vasoactive-inotropic score

^†^Defined as arrhythmia, heart failure or ischaemic heart disease

^‡^Dobutamine, levosimendan, milrinone or adrenalin

Clinical characteristics during TTE are shown in Additional file 1: Table S2.

The feasibility for echocardiographic measurements was limited by technical constraints, resulting in inability to use M-Mode in images from other manufacturers than GE as well as one centre routinely measuring colour Doppler tissue velocities, rather than pulsed wave, making s´ only available for 80 patients (53%). With these difficulties in mind the feasibility was 84%, 71%, 77%, and 77% for LVEF, LVLS, MAPSE, and LV-LWFS, respectively. Interobserver variability measured as the intraclass correlation coefficient was 0.968, 0.984, 0.976, 0.949 and 0.937 for LVEF, s´, LVLS, MAPSE and LWFS, respectively.

### Myocardial injury, organ support-free days and 30-day mortality

More impaired values of LVLS, MAPSE and LV-LWFS, but not LVEF and s´ were observed in patients with myocardial injury (Table 2). Patients with myocardial injury had fewer CRRT-free days. However, OSFD and mortality did not differ between groups.

**Table 2:** Echocardiographic parameters and clinical outcomes for all patients and stratified by myocardial injury

	All *n* = 152	No myocardial injury *n* = 76	Myocardial injury *n* = 76	*p*
LVEF % (IQR), *n* = 127	50 (41–57)	51 (45–58)	48 (38–55)	0.09
s´ cm/s (IQR), *n* = 80	8.3 (6.5–10.6)	9.5 (7.3–11.4)	8.0 (6.4–9.5)	0.09
LVLS % (IQR), *n* = 107	− 13.0 (− 16.4 to − 10.0)	− 14.2 (− 17.4 to − 10.8)	− 12.4 (-14.4 to − 9.3)	0.02
MAPSE mm (IQR), *n* = 122	9 (8–12)	11 (8–13)	9 (6–10)	< 0.001
LV-LWFS % (IQR), *n* = 121	10.5 (8.6–12.6)	11.6 (9.4–14.0)	10.0 (7.4–11.7)	< 0.001
ICU-free days (IQR)	21 (0–27)	22 (6–27)	18 (0–26)	0.07
Vasopressors/inotropes-free days (IQR)	26 (7–28)	27 (16–28)	24 (0–28)	0.06
Mechanical ventilation-free days (IQR)	23 (0–30)	25 (8–30)	22 (0–30)	0.19
CRRT in ICU *n* (%)	46 (31)	18 (26)	26 (35)	0.24
CRRT-free days (IQR)	30 (14–30)	30 (20–30)	27 (0–30)	0.04
Organ support-free days (IQR)	23 (0–28)	24 (7–28)	21 (0–28)	0.13
ICU mortality n (%)	28 (18)	10 (14)	16 (21)	0.27
30-day mortality *n* (%)	32 (21)	12 (17)	18 (24)	0.31

Myocardial injury defined as high-sensitivity troponin T ≥ 45 ng/L on ICU admission

*LVEF*: left ventricular ejection fraction. s´: peak systolic tissue Doppler velocity measured at the mitral annulus. *LVLS*: left ventricular longitudinal strain. MAPSE: mitral annular plane systolic excursion. *LV-LWFS*: left ventricular longitudinal wall fractional shortening. *CRRT*: continuous renal replacement therapy

In multivariable analyses (Table 3), MAPSE and LV-LWFS were independently associated with myocardial injury, whereas LVEF, s´ and LVLS were not.

**Table 3:** Independent relationship between echocardiographic variables and myocardial injury

	Model with LVEF	Model with s’	Model with LVLS	Model with MAPSE	Model with LV-LWFS
LVEF aOR (CI)	0.97 (0.93–1.01) *p* = 0.10	–	–	–	–
s´ aOR (CI)	–	0.93(0.76–1.16) *p* = 0.53	–	–	–
LVLS aOR (CI)	–	–	1.10 (0.98–1.24) *p* = 0.11	–	–
MAPSE aOR (CI)	–	–	–	0.84 (0.70–0.99) *p* = 0.04	–
LV-LWFS aOR (CI)	–	–	–	–	0.85 (0.73–0.99) *p* = 0.04
Age	1.07 (1.03–1.11) *p* = 0.001	1.04 (0.99–1.09) *p* = 0.10	1.06 (1.01–1.10) *p* = 0.01	1.06 (1.01–1.10) *p* = 0.01	1.06 (1.01–1.10) *p* = 0.01
Cardiac disease†	0.77 (0.31–1.91) *p* = 0.58	0.76 (0.26–2.23) *p* = 0.61	0.71 (0.25–1.98) *p* = 0.51	0.83 (0.32–2.13) *p* = 0.70	0.82 (0.32–2.11) *p* = 0.68
SOFA	0.96 (0.83–1.10) *p* = 0.56	0.97 (0.82–1.15) *p* = 0.75	1.12 (0.95–1.33) *p* = 0.19	1.00 (0.86–1.16) *p* = 0.99	0.99 (0.86–1.15) *p* = 0.93
SAPS 3	1.02 (0.99–1.05) *p* = 0.30	1.00 (0.98–1.03) *p* = 0.81	1.01 (0.97–1.04) *p* = 0.67	1.03 (0.99–1.07) *p* = 0.20	1.03 (0.99–1.07) *p* = 0.16
Creatinine	1.00 (1.00–1.01) *p* = 0.02	1.00 (1.00–1.01) *p* = 0.13	1.01 (1.00–1.01) *p* = 0.02	1.00 (1.00–1.01) *p* = 0.02	1.00 (1.00–1.01) *p* = 0.02
RV systolic dysfunction‡	1.34 (0.54–3.33) *p* = 0.53	1.42 (0.43–4.66) *p* = 0.56	1.45 (0.51–4.10) *p* = 0.49	1.03 (0.39–2.75) *p* = 0.96	1.08 (0.41–2.81) *p* = 0.88

Echocardiographic variables (either LVEF, s´, LVLS, MAPSE or LV-LWFS) were included in 5 separate multivariable models adjusted for age, previous cardiac disease, SOFA score, SAPS3 score, creatinine and RV systolic dysfunction. Myocardial injury defined as high-sensitivity troponin T ≥ 45 ng/L on ICU admission. Data are presented as adjusted odds ratio (aOR) (95% confidence interval)

*LVEF* left ventricular ejection fraction. s´: peak systolic tissue Doppler velocity measured at the mitral annulus. *LVLS* left ventricular longitudinal strain. *MAPSE* mitral annular plane systolic excursion. LV-LWFS: left ventricular longitudinal wall fractional shortening. CRRT: continuous renal replacement therapy. SOFA: Sequential Organ Failure Assessment, score at admission. *SAPS 3* Simplified Acute Physiology Score 3, score at admission

^†^Pre-existing cardiac disease, defined as arrhythmias, heart failure or ischaemic heart disease, or any combination of these

^‡^Defined as FWS > -20%, TAPSE < 17 mm, FAC < 35%, tissue or colour s´ < 9.5 cm/s or < 6.0 cm/s, respectively, or (RV/LV) area ratio > 0.66 with concurrent paradoxical septal motion

### Sensitivity analysis

A sensitivity analysis was conducted excluding patients with atrial fibrillation at the time of TTE (*n* = 33). In this population, MAPSE and LV-LWFS were significantly decreased among patients with myocardial injury, however their independent effect was not preserved in the multivariable analysis (Additional file 1: Tables S3, S4).

## Discussion

We demonstrate that MAPSE and LV-LWFS, but not LVEF, s´ and LVLS, are independently associated with myocardial injury in patients with septic shock.

Myocardial injury was chosen as the primary outcome with the intention to establish a relationship between echocardiography variables and biochemical markers of tissue injury, as a possible explanation for poor clinical outcomes. The demonstration of a relationship between echocardiographic markers of systolic function and myocardial injury is a relevant pathophysiological finding and supports the use of a combined biomarker-imaging approach for defining sepsis-induced myocardial dysfunction.

When considered in isolation, LVLS, MAPSE and LV-LWFS, but not LVEF and s´, were associated with myocardial injury on ICU admission. After adjusting for age, pre-existing cardiac disease, SAPS 3 score, RV systolic dysfunction, SOFA score and creatinine on admission, only MAPSE and LV-LWFS maintained a statistically significant association with myocardial injury. LVLS, another echocardiographic measure of longitudinal contractility was more impaired among patients with myocardial injury, but the relationship was not statistically significant after multivariable adjustment. Nevertheless, it may be prudent to keep in mind that the effect size for LVLS may be clinically meaningful and was in the same direction as MAPSE and LV-LWFS. Collectively our findings indicate LV variables reflecting longitudinal displacement are of prognostic significance for myocardial injury and that the other systolic function variables reflect different aspects of LV function. These longitudinal parameters seem to be of less importance among patients without AF. Our results strengthen the recent PRICES expert consensus recommendation [19] for reporting cardiac rhythm at the time of echocardiography and to collect additional information on s’, MAPSE and LV strain for evaluation of LV systolic function.

While we cannot demonstrate a causal relationship, the association between decreased longitudinal contractility and myocardial injury raises the question of why longitudinal function appears to be more affected and if their effect on myocardial injury may be modified. In patients with increased cardiovascular risk, myocardial deformation in the longitudinal plane has important prognostic implications even when LVEF is preserved [22]. Among healthy subjects and patients with decreased LV function, atrioventricular plane longitudinal movement has been shown to be the primary contributor to LV pump function, accounting for 60% of stroke volume [23]. We can only speculate as to whether the septic heart is even more dependent on this process that is reflected biochemically as cardiac enzyme leakage. Nevertheless, decreased MAPSE and LV-LWFS did not translate into poorer clinical outcomes with regard to the need for organ support or mortality at day 30. The lack of a statistical signal confirming an association between early echocardiographic assessment in the ICU and the need for organ support is consistent with a pattern of non-conclusive findings regarding echocardiography in previous literature [24, 25]. In addition, when our data were subjected to analysis using a 3-knot cubic spline function (data not shown), we could not reproduce the results recently reported by Dugar and coworkers [5] that showed a U-shaped relationship between LVEF and mortality. Although we stress that our study was not powered to detect differences in the need for organ support and 30-day mortality, we note that all LV parameters were more impaired in patients with myocardial injury, and patients with myocardial injury had poorer organ function outcomes (Table 2). Surprisingly, the contribution of RV dysfunction appeared to be less important in our model. Therefore, a combination of myocardial injury and impaired LV longitudinal contractility may be a relevant phenotype for future diagnostic and therapeutic research.

Our study has some important limitations. Our data were drawn from two different repositories designed for research. Although the inclusion criteria, echocardiography protocols and baseline patient characteristics were almost identical, we cannot exclude that some differences in management may have occurred. In the later repository, data were collected from a multi-centre study with a majority from 2 centres in Sweden (65% from the coordinating centre) and we cannot exclude country to country differences in management. Nevertheless, all patients were included using septic shock criteria and echocardiography was conducted within 24 h of admission, and all patients were treated according to current guidelines. We measured longitudinal strain from a single view, in contrast to the three views recommended for global longitudinal strain (GLS). The feasibility of GLS is generally poor in patients with septic shock [12] and a single view longitudinal strain has good agreement with GLS and is sufficient for bedside examination [26]. Our study does not discount the value of GLS in the septic shock setting, and it may be possible that the use of GLS, when viable, may be more prognostically informative than LVLS. MAPSE and LV-LWFS are mathematically coupled, and therefore it is not surprising that both parameters had almost identical effect sizes for the outcome. Our results demonstrate the robustness of these related parameters, and that there is no advantage of LV-LWFS over MAPSE for the prediction of myocardial injury. When excluding patients with AF, the prognostic relationship was lost in the multivariable analysis. This may be due to a true lack of effect, a Type II error, or that patients with AF are more dependent on longitudinal function. This finding remains to be confirmed in larger cohorts of patients with and without AF. Our choice of hsTnT cut-off to indicate myocardial injury was arbitrary since there is no currently accepted definition for myocardial injury in sepsis. Using the definition of acute myocardial injury, hsTnT > 14 ng/L and > 20% dynamic change according to the 4th Universal Definition of Myocardial Infarction [27], was deemed unsuitable since 89% of the cohort fulfilled these criteria. The choice of 45 ng/L as a cut-off was based on median values in previous similar populations [15, 16] and is approximately three times the upper reference limit of a normal population and thus more likely to reflect true tissue injury. Although we endeavoured to have simultaneous measurements, we cannot exclude that a delay between hsTnT sampling and echocardiography may have occurred in some patients.

Although no formal assessment was made to exclude acute myocardial infarction (AMI) on admission, we found that two patients suffered AMI during the course of their ICU stay. The study was also underpowered to detect changes in some secondary outcomes, therefore we have made very cautions interpretations regarding OSFD and mortality. We did not design the study to take into account changing trajectories of echocardiographic parameters over the course of ICU stay. We cannot exclude that the echocardiographic abnormalities pre-date ICU admission and mitigated this by considering pre-existing cardiac disease in our analyses. While we were unable to demonstrate a statistically significant independent association with organ failure, future studies should explore this relationship in larger cohorts, taking into account temporal echocardiographic changes.

## Conclusions

The longitudinal systolic function parameters MAPSE and LV-LWFS but not other LV systolic function parameters, are independently associated with myocardial injury in critically ill patients with septic shock. Their relationship with clinical outcomes could not be confirmed and should be explored in future studies.

### Supplementary Information

**Additional file 1: Table S1.** Comparison of the Septic Heart cohort and the Sepsis in the ICU 2 cohort. **Table S2.** Patient characteristics at the time of echocardiography for all patients and stratified by myocardial injury. **Table S3.** Sensitivity analysis excluding patients with atrial fibrillation. Echocardiographic parameters for all patients and stratified by myocardial injury. **Table S4. **Sensitivity analysis excluding patients with atrial fibrillation. Independent relationship between echocardiographic variables and myocardial injury. Echocardiographic variables (either LVEF, s´, LVLS, MAPSE or LV-LWFS) were included in 5 separate multivariable models adjusted for age, previous cardiac disease, SOFA score, SAPS3 score, creatinine and RV systolic dysfunction. **Table S5.** PRICES checklist items.

## Data Availability

The data from this study will be made available after publication, upon application to the corresponding author and within the terms of the Global Data Protection Regulation and the Swedish Patient Data Law (2008:355). To avoid the possibility of identifying individual cases, detailed data are not given in the paper but may be requested from the corresponding author.

## Abbreviations

LVLS: Left ventricular longitudinal strain
LVEF: Left ventricle ejection fraction
MAPSE: Mitral annular plane systolic excursion
LV-LWFS: Left ventricle longitudinal wall fractional shortening
s´: Peak tissue Doppler velocity at the mitral valve
OSFD: Organ support-free days
TTE: Transthoracic echocardiography
ICU: Intensive care unit
SAPS 3: Simplified acute physiology score 3
SOFA: Sequential organ failure assessment
NTproBNP: N-terminal pro B-type natriuretic peptide
SCM: Septic cardiomyopathy
LV: Left ventricle
VL: Ventricle length
HsTnT: High-sensitivity troponin T
SICU-2: Sepsis in the ICU-2
STROBE: Strengthening the reporting of observational studies in epidemiology
DICOM: Digital imaging and communications in medicine
FPS: Frames per second
LVSD: Left ventricular systolic dysfunction
PRICES: Preferred Reporting Items for Critical care Echocardiography Studies
EDTA: Ethylenediaminetetraacetic acid
IQR: Interquartile range
CFS: Clinical frailty scale
CRRT: Continuous renal replacement therapy
OR: Odds ratio
CI: Confidence interval
aOR: Adjusted odds ratio
GLS: Global longitudinal strain
AMI: Acute myocardial infarction
mmHg: Millimetres of mercury
cmH_2_O: Centimetres of water
VIS: Vasoactive-inotropic score
PaO_2_: Arterial partial pressure of oxygen
FiO_2_: Fraction of inspired oxygen
kPa: Kilo Pascal
