# Supplementary material for: Association between left ventricular systolic function parameters and myocardial injury, organ failure and mortality in patients with septic shock
Source: Ann Intensive Care. 2024 Jan 18;14:12. doi: 10.1186/s13613-023-01235-5 (PMC10796855; doi:10.1186/s13613-023-01235-5)
Supplement: Supplementary file 1 — Additional file 1: Table S1. Comparison of the Septic Heart cohort and the Sepsis in the ICU 2 cohort. Table S2. Patient characteristics at the time of echocardiography for all patients and stratified by myocardial injury. Table S3. Sensitivity analysis excluding patients with atrial fibrillation. Echocardiographic parameters for all patients and stratified by myocardial injury. Table S4. Sensitivity analysis excluding patients with atrial fibrillation. Independent relationship between echocardiographic variables and myocardial injury. Echocardiographic variables (either LVEF, s´, LVLS, MAPSE or LV-LWFS) were included in 5 separate multivariable models adjusted for age, previous cardiac disease, SOFA score, SAPS3 score, creatinine and RV systolic dysfunction. Table S5. PRICES checklist items. [file 13613_2023_1235_MOESM1_ESM.docx]

**Table S1.** Comparison of the Septic Heart cohort and the Sepsis in the ICU 2 cohort.

|  | **Septic Heart** n = 44 | **Sepsis in the ICU 2** n = 108 | **p** |
| --- | --- | --- | --- |
| **Age**  years (IQR) | 65  (56-73) | 72 (59-76) | 0.06 |
| **Sex**  n (%) | 27  (61) | 57  (53) | 0.33 |
| **Body mass index**  kg/m^2^ (IQR) | 24.6  (22.3-31.8) | 28.3  (23.9-32.5) | 0.03 |
| **Pre-existing cardiac disease**†  n (%) | 21  (48) | 38  (35) | 0.15 |
| **SOFA**  score (IQR) | 10  (9-12) | 9  (7-12) | 0.01 |
| **SAPS 3**  score (IQR) | 72  (61-81) | 67  (57-76) | 0.12 |
| **Haemoglobin**  g/L (IQR) | 107  (96-114) | 97  (79-109) | 0.002 |
| **Creatinine**  μmol/L (IQR) | 127  (85-191) | 156  (100-230) | 0.04 |
| **Lactate**  mmol/L (IQR) | 3.4  (1.9-4.7) | 3.2  (2.1-4.8) | 0.86 |
| **hsTnT**  ng/L (IQR) | 40  (23-128) | 52  (25-99) | 0.97 |
| **Inotropes**‡  n (%) | 29  (66) | 35 (32) | <0.001 |
| **Myocardial injury** | 20  (46) | 56  (57) | 0.12 |
| **Mechanical ventilation at echocardiography** | 29  (66) | 59  (55) | 0.20 |

Myocardial injury defined as high-sensitivity troponin T ≥45 ng/L on ICU admission.

†Defined as arrhythmia, heart failure or ischaemic heart disease.

‡Dobutamine, levosimendan, milrinone or adrenalin.

SOFA: Sequential Organ Failure Assessment. SAPS 3: Simplified Acute Physiology Score 3. HsTnT: high-sensitive Troponin T.

**Table S2.** Patient characteristics at the time of echocardiography for all patients and stratified by myocardial injury.

|  | **All** n = 152 | **No myocardial injury** n = 71 | **Myocardial injury** n = 76 |
| --- | --- | --- | --- |
| **Atrial fibrillation** n (%) | 33 (22) | 8 (24) | 24 (76) |
| **Other non-sinus rhythms** n (%) | 6 (4) | 4 (67) | 2 (33) |
| **Heart rate** bpm (IQR) | 94 (85-110) | 92 (85-107) | 97 (85-114) |
| **Systolic arterial pressure** mmHg (IQR), n = 148 | 110 (99-121) | 109 (99-121) | 110 (94-122) |
| **Diastolic arterial pressure** mmHg (IQR), n = 148 | 54 (48-60) | 55 (46-60) | 53  (50-60) |
| **Mean arterial pressure** mmHg (IQR), n = 148 | 73 (66-80) | 73 (66-80) | 73 (67-79) |
| **Cardiac index** L/min/m2 (IQR), n = 62 | 3.6 (3.0-4.7) | 4.0 (3.3-4.7) | 3.4 (2.4-4.6) |
| **Systemic vascular resistance index** s*m^2^/cm^5^ (IQR), n = 54 | 1 300 (933-1 907) | 1 240 (990-1 785) | 1 310 (850-960) |
| **Central venous pressure** cmH_2_O (IQR), n = 78 | 13 (8-17) | 13 (9-18) | 14 (7-16) |
| **Mechanically ventilated** n (%) | 88 (58) | 40 (46) | 44 (50) |
| **Peak inspiratory pressure** cmH_2_O (IQR) | 22 (19-26) | 24 (20-28) | 21 (19-25) |
| **Driving pressure** cmH_2_O (IQR) | 12 (10-15) | 12 (11-16) | 12 (10-14) |
| **Peak end-expiratory pressure** cmH_2_O (IQR) | 10 (8-12) | 10 (8-13) | 10 (8-12) |
| **PaO_2_/FiO_2_** ratio (IQR), n = 134 | 26.7 (20-40) | 25.9 (17.9-40.2) | 28.1 (21.0-40.0) |
| **PaO2** kPa (IQR), n = 140 | 11.1 (9.8-13.3) | 10.5 (9.4-12.7) | 11.6 (10.2-13.8) |

Myocardial injury defined as high-sensitivity troponin T ≥45 ng/L on ICU admission.

PaO_2_: arterial partial pressure of oxygen. FiO_2_: fraction of inspired oxygen.

**Table S3.** Sensitivity analysis excluding patients with atrial fibrillation. Echocardiographic parameters for all patients and stratified by myocardial injury.

|  | **All** n = 119 | **No myocardial injury** n = 63 | **Myocardial injury** n = 52 | **p** |
| --- | --- | --- | --- | --- |
| **LVEF** % (IQR), n = 99 | 50 (44-57) | 51 (45-58) | 49 (39-57) | 0.25 |
| **s´** cm/s (IQR), n = 62 | 9.0 (6.9-11.3) | 9.7 (7.7-11.8) | 8.8 (6.7-9.9) | 0.13 |
| **LVLS** % (IQR), n = 89 | -13.4 (-16.8- -10.9) | -14.4 (-17.5- -11.3) | -13.1 (-15.6- -9.8) | 0.13 |
| **MAPSE** mm (IQR), n = 94 | 10  (8-12) | 11 (8-13) | 9 (8-10) | 0.05 |
| **LV-LWFS** % (IQR), n = 93 | 11.0  (9.4-13.1) | 11.6 (9.5-14.4) | 10.5 (8.8-11.9) | 0.04 |

Myocardial injury defined as high-sensitivity troponin T ≥45 ng/L on ICU admission.

LVEF: Left Ventricular Ejection Fraction. s´: peak systolic tissue Doppler velocity measured at the mitral annulus. LVLS: Left Ventricular Longitudinal Strain. MAPSE: Mitral Annular Plane Systolic Excursion. LV-LWFS: Left Ventricular Longitudinal Wall Fractional Shortening. CRRT: Continuous Renal Replacement Therapy.

**Table S4.** Sensitivity analysis excluding patients with atrial fibrillation. Independent relationship between echocardiographic variables and myocardial injury. Echocardiographic variables (either LVEF, s´, LVLS, MAPSE or LV-LWFS) were included in 5 separate multivariable models adjusted for age, previous cardiac disease, SOFA score, SAPS3 score, creatinine and RV systolic dysfunction.

|  | **Model with LVEF** | **Model with s’** | **Model with LVLS** | **Model with MAPSE** | **Model with LV-LWFS** |
| --- | --- | --- | --- | --- | --- |
| **LVEF**  aOR (CI) | 0.96 (0.91-1.01) 0.09 |  |  |  |  |
| **s´**  aOR (CI) |  | 0.87 (0.67-1.12) 0.28 |  |  |  |
| **LVLS**  aOR (CI) |  |  | 1.08 (0.95-1.23) 0.23 |  |  |
| **MAPSE**  aOR (CI) |  |  |  | 0.90 (0.74-1.11) 0.33 |  |
| **LV-LWFS**  aOR (CI) |  |  |  |  | 0.89 (0.75-1.06) 0.21 |
| **Age** | 1.06 (1.02-1.11) 0.004 | 1.02 (0.97-1.07) 0.38 | 1.06 (1.01-1.10) 0.02 | 1.05 (1.01-1.10) 0.02 | 1.05 (1.01-1.10) 0.02 |
| **Cardiac disease**† | 0.84 (0.30-2.35) 0.73 | 1.12 (0.31-4.14) 0.86 | 0.74 (0.24-2.30) 0.60 | 0.93 (0.31-2.76) 0.70 | 0.94 (0.32-2.79) 0.91 |
| **SOFA** | 0.91 (0.78-1.07) 0.26 | 0.99 (0.82-1.20) 0.94 | 1.09 (0.91-1.32) 0.35 | 0.97 (0.82-1.14) 0.70 | 0.97 (0.82-1.14) 0.68 |
| **SAPS 3** | 1.03 (0.99-1.07) 0.15 | 1.00 (0.97-1.03) 0.86 | 1.01 (0.97-1.05) 0.62 | 1.04 (0.99-1.08) 0.12 | 1.04 (0.99-1.09) 0.09 |
| **Creatinine** | 1.00 (1.00-1.01) 0.04 | 1.00 (1.00-1.01) 0.51 | 1.00 (1.00-1.01) 0.07 | 1.00 (1.00-1.01) 0.05 | 1.00 (1.00-1.01) 0.06 |
| **RV systolic dysfunction**‡ | 0.95 (0.35-2.59) 0.91 | 0.95 (0.24-3.73) 0.94 | 1.26 (0.41-3.90) 0.69 | 1.09 (0.38-3.12) 0.88 | 1.08 (0.39-3.01) 0.89 |

Myocardial injury defined as high-sensitivity troponin T ≥45 ng/L on ICU admission. Data are presented as adjusted odds ratio (aOR) (95% confidence interval).

†Pre-existing cardiac disease, defined as arrhythmias, heart failure or ischaemic heart disease, or any combination of these. ‡Defined as FWS >-20%, TAPSE <17 mm, FAC <35%, tissue or colour s´ <9.5 cm/s or <6.0 cm/s, respectively, or (RV/LV) area ratio >0.66 with concurrent paradoxical septal motion.

LVEF: Left Ventricular Ejection Fraction. s´: peak systolic tissue Doppler velocity measured at the mitral annulus. LVLS: Left Ventricular Longitudinal Strain. MAPSE: Mitral Annular Plane Systolic Excursion. LV-LWFS: Left Ventricular Longitudinal Wall Fractional Shortening. CRRT: Continuous Renal Replacement Therapy. SOFA: Sequential Organ Failure Assessment, score at admission. SAPS 3: Simplified Acute Physiology Score 3, score at admission.

**Supplementary table 5.** PRICES checklist items.

| PRICES CHECKLIST ITEMS | LV systolic function | RV systolic function |
| --- | --- | --- |
| **Research vs Clinical Study** |  |  |
| Research Study | Yes | Yes |
| Clinical Study | Yes | Yes |
| **Study Information** |  |  |
| Specific study type | Yes | Yes |
| State study design | Yes | Yes |
| Report Sample Size | Yes | Yes |
| **Patient Information** |  |  |
| Age | Yes | Yes |
| Gender | Yes | Yes |
| Height and Weight (or BMI) | Yes | Yes |
| *Comorbidities* |  |  |
| Ischaemic heart disease* | Yes | Yes |
| Atrial fibrillation* | Yes | Yes |
| Hypertension | Not provided | Not provided (NE) |
| HFpEF | Not provided | Not provided (NE) |
| HFrEF | Not provided | Not provided |
| Pacemaker implant present | Not provided | Not provided |
| COPD or pulmonary hypertension** | Not provided (NE) | Not provided |
| CKD or hemodialysis | Not provided (NE) | Not provided (NE) |
| **Echocardiography information** |  |  |
| Type of echo (TTE/TEE) | Yes | Yes |
| Data collected at end-expiration | Not provided | Not provided |
| No. of beats used for averaging | Yes | Yes |
| Vendor of ultrasound machine | Yes | Yes |
| Airway pressure trace displayed on screen | Not provided (NE) | Not provided (NE) |
| **Clinical information at time of echo** |  |  |
| *Ventilation* |  |  |
| Mode of ventilation | Yes | Yes |
| Tidal volume | Yes | Yes |
| Plateau pressure | Yes (NE) | Yes |
| PEEP | Yes (NE) | Yes |
| *Haemodynamics* |  |  |
| Cardiac rhythm and heart rate | Yes | Yes |
| Blood pressure | Yes | Yes |
| Inotropes, vasopressors and doses | Yes | Yes |
| *Reliability* |  |  |
| Feasibility of echo | Yes | Yes |
| Intraobserver variability | Not provided (NE) | No |
| Interobserver variability | Yes (NE) | No |
| Indicate if observer blinded to treatment | Yes (NE) | Yes |
| **Statistics** |  |  |
| Sample size calculation | Yes | Yes |
| Statistician blinded to treatment | Yes | Yes |
| Address confounders if applicable | N/A (NE) | N/A |
| Internal validation, if applicable | N/A | N/A |
| **LV systolic function indices** |  |  |
| LV ejection fraction | Yes | N/A |
| Tissue Doppler S’ velocity | Yes (NE) | N/A |
| Mitral annular plane systolic excursion (MAPSE) | Yes (NE) | N/A |
| LV strain or strain rate | Yes (NE) | N/A |
| **LV size** |  |  |
| LV end-diastolic diameter/volume | No | N/A |
| **Other functional indices to aid interpretation** |  |  |
| Cardiac output | Yes | N/A |
| Stroke volume | Not provided | Not provided |
| Any heart valve dysfunction | Not provided | Not provided |
| **RV systolic function indices** |  |  |
| Tricuspid annular plane systolic excursion (TAPSE) | N/A | Yes |
| RV fractional area change | N/A | Yes |
| Tissue Doppler s’ velocity | N/A | Yes |
| RV strain or strain rate | N/A | Yes |
| **RV size and wall thickness** |  |  |
| RV end diastolic diameter or area | N/A | Not provided |
| RV:LV end diastolic area ratio | N/A | Yes |
| RV wall thickness | N/A | Not provided |
| **Other functional indices to aid interpretation** |  |  |
| PFO or other shunt(s) | Not provided | Not provided |
| Pericardial effusion | Not provided | Not provided |
| Paradoxical septal motion | N/A | Yes |
| Interatrial septal bowing | Not provided | Not provided |
| IVC diameter | N/A | Not provided |

*Recorded as preexisting cardiac disease and defined as arrythmias, heart failure or ischaemic heart disease, or any combination of these. NE: Non-essential item as designated by the PRICES statement.
